# In Situ Cleaning of Bead Surfaces by Utilizing Continuous High-Power Laser Scanning

**DOI:** 10.3390/ma18071423

**Published:** 2025-03-23

**Authors:** Jun Xiao, Ruikun Liu, Xinyu Ge, Weixing Sheng, Shengnan Gai, Shujun Chen

**Affiliations:** 1 College of Mechanical and Energy Engineering, Beijing University of Technology, Beijing 100124, China; jun.xiao@bjut.edu.cn (J.X.); lrk0115@163.com (R.L.); gxy32@live.cn (X.G.); shengweixing@emails.bjut.edu.cn (W.S.); sjchen@bjut.edu.cn (S.C.); 2 Engineering Research Center of Advanced Manufacturing Technology for Automotive Components, Ministry of Education, Welding Equipment R&D Center, Beijing University of Technology, Beijing 100124, China

**Keywords:** aluminum alloy, in situ cleaning, oscillating laser, formation quality

## Abstract

In situ cleaning of the weld area on a substrate or weld beads is performed by adjusting power and the laser scanning speed using a conventional continuous high-power laser typically employed for welding or deposition. This process facilitates the removal of contaminants (e.g., oil residues, metal oxides, and post-weld black ash) and enables efficient planning and execution of the welding process. The influence of varying laser scanning power on the cleaning efficacy of a 6061 aluminum alloy substrate was examined. The findings revealed that, as the laser scanning power increased from 1200 W to 3900 W, the cleaning effectiveness on the aluminum alloy surface initially improved and then diminished. At lower laser scanning power levels, the energy was insufficient to evaporate and disintegrate the surface contaminants, whereas excessively high laser power tended to cause secondary burns and oxidation on the material surface. Additionally, excessively high laser scanning speeds reduced the overlap of the scanning area, thereby compromising the cleaning results. The proposed surface cleaning method, utilizing a standard continuous high-power laser, demonstrated satisfactory cleaning performance through visual inspection, oxide detection, and analysis of porosity defects in the weld beads produced post-cleaning. Pre-laser scanning and cleaning significantly reduced the incidence of porosity and enhanced weld quality. This method eliminates the need for a specialized laser cleaning system powered by short-pulse lasers and can serve as a supplementary approach to conventional cleaning methods or in situations where alternative methods are unavailable. Oscillating laser scanning can also be applied to clean curved weld surfaces, effectively removing contaminants and oxides from the deposited weld surface, which is particularly beneficial for multi-layer, multi-pass welding or additive manufacturing processes.

## 1. Introduction

Aluminum alloy materials are extensively utilized in aerospace and various other industries. However, welding and additive manufacturing processes for aluminum alloy components are often challenged by high porosity and crack susceptibility [1,2]. Aluminum alloys are particularly prone to oxidation, and the presence of oxide films and other surface contaminants can significantly increase porosity and compromise weld quality [3,4,5]. Additionally, the welding or additive fusion wire process generates a substantial amount of metal oxides, often manifesting as post-weld “black ash.” Subsequent production processes can introduce serious defects, including porosity, cracks, and poor interlayer bonding [6,7,8]. Consequently, surface cleaning of the workpiece prior to welding is typically a mandatory step in the welding process specification. Traditional cleaning methods include mechanical and chemical cleaning. Mechanical cleaning involves the conversion of external energy, such as high-speed impact or mechanical friction, to remove surface contaminants. However, mechanical cleaning can reduce the volume of the additively shaped weld, thereby increasing energy consumption and decreasing the efficiency of additive fusion wire deposition [9]. Chemical cleaning employs cleaning agents to chemically treat the material surface, but improper handling can pose health hazards and cause irreversible environmental pollution [10]. Furthermore, both mechanical and chemical cleaning are typically performed offline, introducing cumbersome cleaning steps and increasing the risk of secondary oxidation in the cleaned area. Additionally, for multi-layer, multi-channel welding or additive manufacturing, the high-temperature weld with a fusion layer surface is more susceptible to oxidation. Mechanical or chemical cleaning must wait until the weld temperature cools to below 60 °C, thereby increasing the waiting time for welding operations and affecting the efficiency of subsequent layer deposition [11,12]. Therefore, to address the limitations of conventional cleaning methods, there is a critical need for a more efficient, environmentally friendly cleaning approach that can be implemented online.

Laser cleaning, which employs high-energy lasers to remove surface contaminants through mechanisms such as photoionization, photoablation, and photothermal elastic vibration, offers advantages including environmental sustainability, high efficiency, and ease of integration with subsequent equipment. These benefits have established laser cleaning as a novel approach in industrial cleaning, leading to its widespread adoption as an advanced cleaning method [13,14,15]. In 1965, A.L. Schawlow introduced the concept of a laser eraser, wherein pulsed laser irradiation was applied to ink on paper, rapidly vaporizing the ink without damaging the paper. This marked the first documented application of lasers in cleaning practices [16]. With ongoing advancements in laser cleaning technology, pulsed lasers have become extensively utilized in surface cleaning applications. J. Chen et al. compared various methods, including sandpaper polishing, acid washing, and laser cleaning, for preparing the surface of a Ti-6Al-4V plate prior to welding. Their findings demonstrated that laser cleaning, when conducted under optimal parameters, effectively removed the oxide film, reduced surface roughness, and resulted in welds with low porosity [17]. Similarly, Z.C. Li et al. investigated the cleaning efficacy of mechanical and laser cleaning methods on TA15 titanium alloy oxide films. Their study revealed that, while mechanical and chemical cleaning methods effectively removed the oxide film, laser cleaning not only achieved comparable results but also enhanced surface properties [18]. Z. Li et al. investigated the enhancement of welding performance and the mitigation of welding defects in titanium alloy components through laser cleaning. Their findings indicated that the oxygen content initially decreased and subsequently increased with higher laser power and scanning frequency, while it decreased with increased cleaning speed [19]. Y. Ren et al. demonstrated that laser cleaning effectively removes impurities and oxides from the surface of AA2024. However, when the energy density exceeds the optimal level, thermal oxidation occurs on the surface, and residual stresses on the aluminum alloy surface are reorganized, resulting in residual tensile stresses [20]. Although pulsed laser cleaning has shown improved effectiveness, the associated equipment is costly. Furthermore, the current industrial application of laser cleaning devices necessitates specialized equipment, which is not easily integrated into conventional continuous laser welding systems for continuous multi-pass, multi-channel welding or additive manufacturing operations. This requirement for alternating between welding and cleaning equipment leads to more complex processes. Additional challenges include low cleaning efficiency and sensitivity to cleaning parameters [21]. Continuous laser cleaning addresses these issues, offering a more economical solution by reducing energy consumption per unit area by 40–60% and being suitable for prolonged continuous operation [22]. However, research on the application of continuous laser cleaning remains limited and inadequately documented. Madhukar et al. examined the effectiveness of a continuous laser and millisecond pulsed laser in removing lacquer layers using a Yb-doped fiber laser. Their results demonstrated that a continuous wave laser can achieve comparable cleaning effects to a millisecond pulsed laser when appropriate cleaning parameters are applied [23]. X. Sun et al. utilized a continuous wave laser to clean the surface coating of AA7075. Their experimental results revealed that the coating cleaning thickness increased with higher laser power density, but excessive power density caused damage to the substrate material [24]. In the aforementioned studies on continuous wave laser cleaning, research has primarily focused on the cleaning efficacy of surface coatings, with limited investigation into the removal of pollutants such as black ash generated during welding operations and the impact on post-welding quality.

In this study, a method for in situ cleaning utilizing continuous wave laser scanning is proposed. This method employs a high-power laser welding heat source for oscillating scanning, with appropriate modulation of laser power and scanning speed. The effectiveness of this approach is analyzed and compared with conventional chemical cleaning methods. Additionally, the impact of various parameters on the quality of weld seam formation post-welding is examined.

## 2. Materials and Methods

### 2.1. Continuous High-Power Laser Scanning System

**Figure 1:**
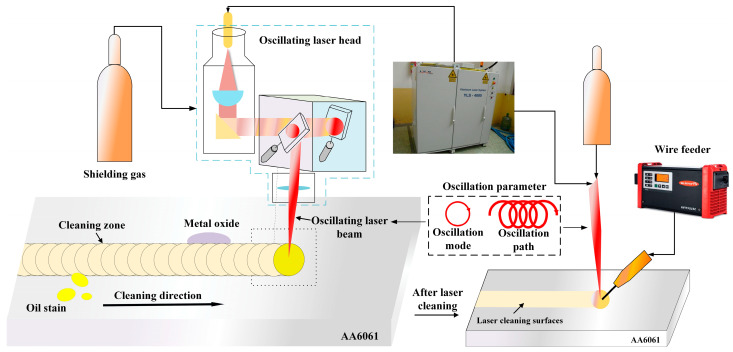
Schematic diagram of the experimental system.

In this experiment, a continuous wave ytterbium-doped fiber laser (YLS-4000) with a maximum output power of 4 kW, manufactured by IPG (Oxford, MA, USA), was employed as the cleaning heat source. The relevant technical parameters are presented in Table 1. The laser beam was transmitted through a 50 μm diameter, 15 m long optical fiber, shaped, and output via an oscillating laser welding head. The cleaning process was conducted using a KUKA six-axis robot (Augsburg, Germany) and its associated control system, which drove the oscillating laser welding head. Auxiliary devices for laser cleaning included water-cooling circuits for cooling the oscillating laser welding head, as well as protective gas systems. The system equipment is illustrated in Figure 1, where the wire feeder was utilized for subsequent laser welding tests.

An experiment was conducted using a 5 mm thick 6061 aluminum alloy as the laser cleaning object, with dimensions of 200 mm × 30 mm × 5 mm. The chemical composition and mechanical properties of the alloy are presented in Table 2 and Table 3. A wire with the same composition as the cleaning object, having a diameter of 1.2 mm, was utilized. The protective gas employed was 99.99% pure argon, with a controlled flow rate of 15 L/min.

### 2.2. Experimental Procedure and Characterization Method

The beam produced by the continuous wave fiber laser generates an oscillating beam through the rapid movement of the vibrating mirror within the oscillating laser welding head. The cleaning-related parameters are detailed in Table 4, and this oscillating motion is superimposed with the robot’s uniform (referred to as the cleaning speed), ensuring that the laser spot’s scanning path covers the entire area to be cleaned. The laser welding process parameters employed in the experiment are presented in Table 5. To examine the morphology and surface quality of the material after oscillating laser cleaning, an Olympus LEXT OLS 4000 laser confocal microscope (Tokyo, Japan) and scanning electron microscope (SEM) were utilized. To accurately assess the removal of oxides, oil, and other contaminants from the material surface, energy-dispersive X-ray spectroscopy (EDS) analysis was conducted using an SU9000 scanning transmission electron microscope (Tokyo, Japan). In addition to laser cleaning, the oxygen content on the material surface after chemical cleaning was measured to serve as a control group for evaluating the laser cleaning effectiveness. The chemical cleaning procedure involved the following steps: first, the material surface was polished with 400-grit sandpaper; second, the sample surface was cleaned with an acetone solution; and finally, the sample was immersed in a 10% volume fraction sodium hydroxide aqueous solution for 5 min, followed by rinsing with clean water and air drying. To minimize secondary contamination of the cleaned surfaces by airborne substances such as moisture, the cleaned, dried, and cooled surfaces were protected using vacuum packaging, thereby eliminating potential influences on the EDS test results. Laser welding experiments were conducted on the cleaned surfaces, which were subsequently cut into 10 mm × 10 mm metallographic specimens using a wire cutter. Porosity was calculated as the ratio of the total cross-sectional area of the porosity to total area of the weld. The impact of the cleaning process on weld grain structure was analyzed using the electron backscatter diffraction (EBSD) technique.

## 3. Results and Discussion

### 3.1. Effect of Laser Power on Material Surface Quality

**Figure 2:**
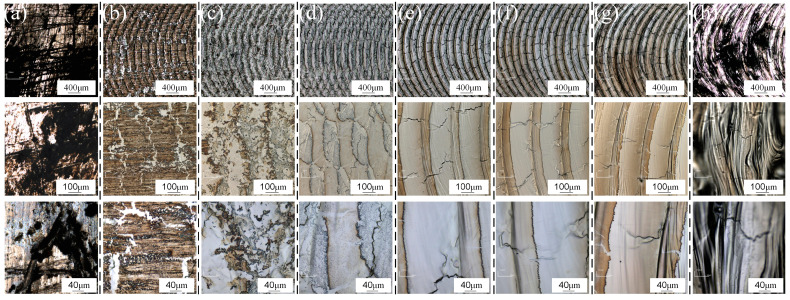
Surface quality under different laser powers: (**a**) unwashed; (**b**) P = 1200 W; (**c**) P = 2400 W; (**d**) P = 2700 W; (**e**) P = 3000 W; (**f**) P = 3300 W; (**g**) P = 3600 W; (**h**) P = 3900 W.

A 6061 aluminum alloy, which had not been subjected to welding contamination for an extended period, was selected for laser cleaning experiments. The laser cleaning speed was maintained at 3.6 m/min, and the remaining parameters are detailed in Table 4. Figure 2 illustrates the surface morphology of the material after cleaning at different laser power levels. When the material is not cleaned, there are a lot of scratches on the surface and black contaminants are attached. At a lower laser power of 1200 W, the high reflectivity of the aluminum alloy surface resulted in a visible white flash during the cleaning process, triggering a high anti-alarm response. Due to the insufficient power density to fully evaporate the surface contaminants, a significant amount of brown metal oxide layer remained observable on the material surface.

With the increase in laser power to P = 2400 W, the broken oxide film becomes observable, revealing the underlying unoxidized aluminum alloy. The scanning path of the oscillating laser can be vaguely discerned; however, the laser power at this stage remains insufficient to achieve an effective cleaning result. When the laser power is increased to P = 2700 W, the oscillating laser scanning pattern becomes more distinct, and a small amount of metal evaporation occurs on the material surface. While the majority of contaminants were removed, a significant number of black particles appear at the edges of the oscillating path. This phenomenon is attributed to the Gaussian distribution of the laser energy, where the central region exhibits higher energy intensity compared to the lower energy levels at the periphery. Although the laser scanning path covers the entire cleaning area, the energy at the edges of the oscillating laser is too low to completely eliminate the contaminants, causing them to accumulate at the sides of the laser path at a consistent cleaning speed.

**Figure 3:**
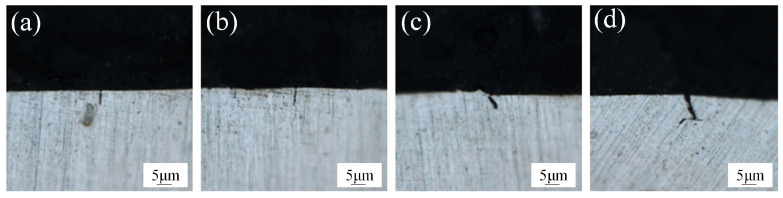
Cracks left after cleaning: (**a**) P = 2700 W; (**b**) P = 3000 W; (**c**) P = 3300 W; (**d**) P = 3600 W.

With a further increase in laser power to P = 3300 W, black pollutant particles are no longer produced on the edge swept by the oscillating laser. The material surface exhibits a silver-white metallic luster, and the oxides on the surface of the 6061 aluminum alloy are thoroughly removed. However, a wave-like morphology, similar to that observed after pulsed laser cleaning, is observed on the scanning edge. This phenomenon is attributed to the rapid flow of molten metal to both sides due to laser recoil following oscillating laser scanning, resulting in the formation of a wave-like shape. Upon further increasing the laser power, it is observed that the surface develops black folds and loses its metallic luster, transitioning from white to black. Analysis indicates that, at a laser power of P = 3900 W, excessive power leads to secondary ablation on the surface of the material surface, resulting in unclear surface morphology, severe oxidation, and poor cleaning effectiveness. As illustrated in Figure 2, when the laser power is increased to 2700 W, microcracks perpendicular to the oscillating laser cleaning path appear. The length and number of these cracks gradually increase with higher laser power. The formation of these cracks is analyzed as follows: first, unlike pulsed laser cleaning, which releases high-density heat in a very short time, continuous wave laser cleaning generates continuous heat on the material surface. Combined with the multiple thermal cycles of the oscillating laser, heat accumulation and temperature gradients induce significant thermal stresses, leading to surface cracks. Second, a thin oxide film forms on the surface of aluminum alloy over time. During laser cleaning, increased laser power can cause the oxide film to rupture, resulting in stress concentration and subsequent crack formation. The depth of cracks under different power levels was subsequently studied, as shown in Figure 3. With increasing laser power, the crack depth gradually increases. At P = 3600 W, flat bubbles are observed at the crack ends, which are presumed to be induced by porosity defects from the die-casting process of the plate. The measured crack depths range from 5 to 10 µm, comparable to the size of minor surface scratches. Laser cleaning does not affect the material’s usability, and these cracks are expected to heal during subsequent welding processes.

**Figure 4:**
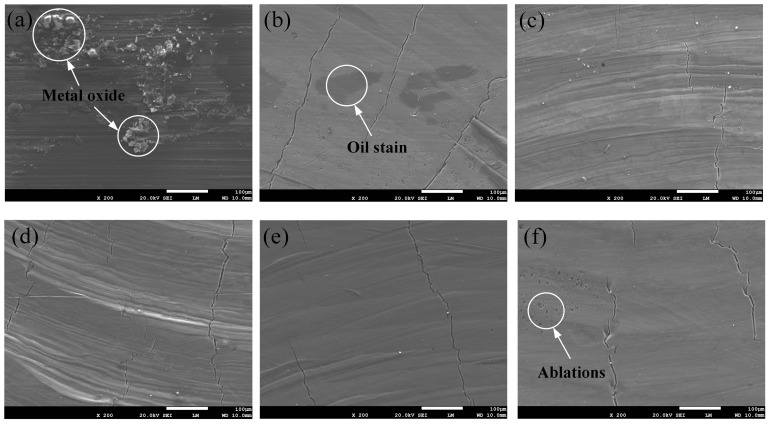
Cleaning effect under different laser powers in SEM: (**a**) unwashed; (**b**) P = 1200 W; (**c**) P = 2700 W; (**d**) P = 3000 W; (**e**) P = 3300 W; (**f**) P = 3600 W.

**Figure 5:**
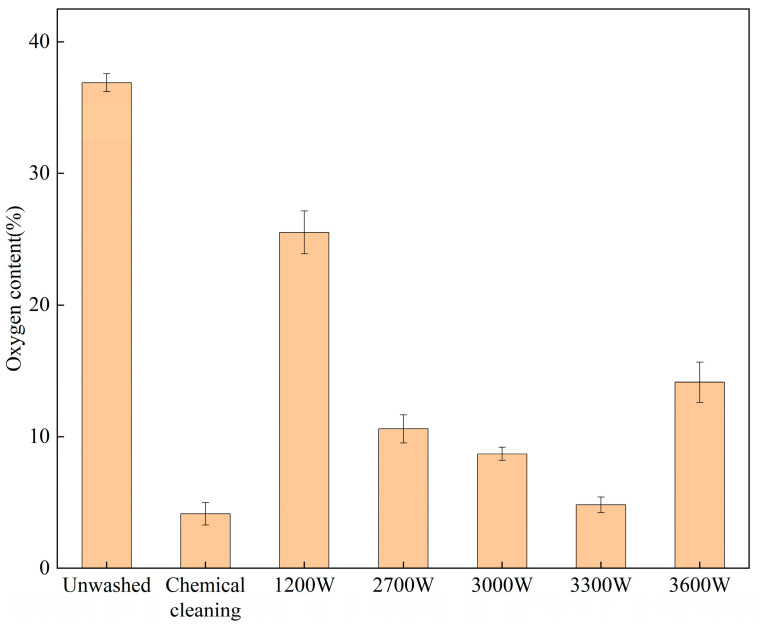
Surface oxygen content under different laser power cleaning treatments.

To evaluate the cleaning effectiveness under different parameters, a method for assessing cleaning effectiveness was established. This method involved observing the presence of any residual contaminants after cleaning using SEM and laser confocal microscopy, as well as analyzing changes in the oxygen content on the surface after cleaning using EDS. As illustrated in Figure 4, SEM images of the surface before cleaning and under different laser cleaning powers are presented. It is evident that when the material surface is not cleaned, it is densely covered with flocculent oxides and oil contaminants. However, when the surface is cleaned with a laser, these flocculent oxides and oil pollutants gradually disappear as the cleaning power increases, leaving no significant traces of residual contaminants on the surface. To further characterize the cleaning effect, the oxygen content of the chemically cleaned surfaces was analyzed using EDS, as shown in Figure 5, for both uncleaned surfaces and surfaces cleaned with different laser powers. The control group, consisting of uncleaned surfaces, exhibited an oxygen content of 37.45%. When the laser power was set to 1200 W, the oxygen content was 23.36%, indicating insufficient fragmentation to remove surface contaminants. As the power increased, the oxygen content on the surface initially increased and then decreased. At a power of 3300 W, the oxygen content was only 5.39%, slightly higher than that after chemical cleaning, representing the optimal cleaning effect. Compared to the control group, the oxygen content was reduced by 85.6%. With a further increase in laser power to 3600 W, the surface experienced slight ablation, and the oxygen content increased to 13.34%.

### 3.2. Effect of Cleaning Speed on Material Surface Quality

**Figure 6:**
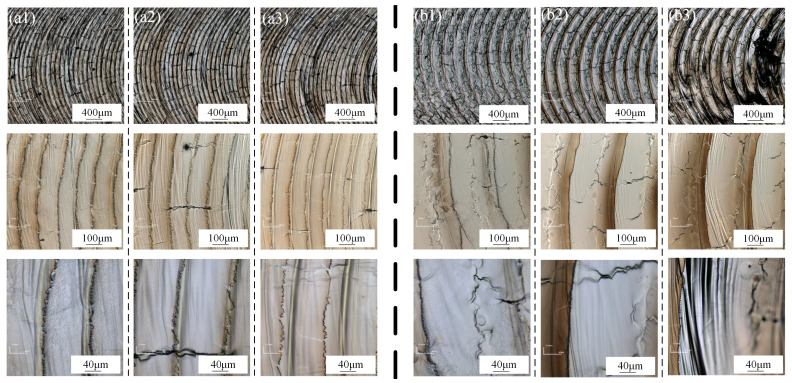
Surface quality under different cleaning speeds: (**a1**) v = 1.8 m/min P = 2700 W; (**a2**) v = 1.8 m/min P = 3000 W; (**a3**) v = 1.8 m/min P = 3300 W; (**b1**) v = 4.5 m/min P = 3000 W; (**b2**) v = 4.5 m/min P = 3300 W; (**b3**) v = 4.5 m/min P = 3600 W.

The cleaning speeds are set at v = 1.8 m/min and v = 4.5 m/min, respectively. Based on the findings from the previous section, the optimal cleaning effect is achieved when the laser power P = 3300 W. Consequently, the optimal cleaning effect is observed near the current parameter with the cleaning power. The surface morphology after cleaning is illustrated in Figure 6.

Similar to pulsed laser cleaning, oscillating laser cleaning also involves the concept of spot overlap rate, which is determined by the spot diameter D (mm), oscillation frequency f (Hz), and cleaning speed v (m/s). The oscillating laser cleaning path overlap rate, denoted as r, represents the proportion of the path scanned by the previous laser that is covered by the subsequent laser. This rate is calculated using the following formula:

(1) $$r=1-\frac{v}{Df}$$

**Figure 7:**
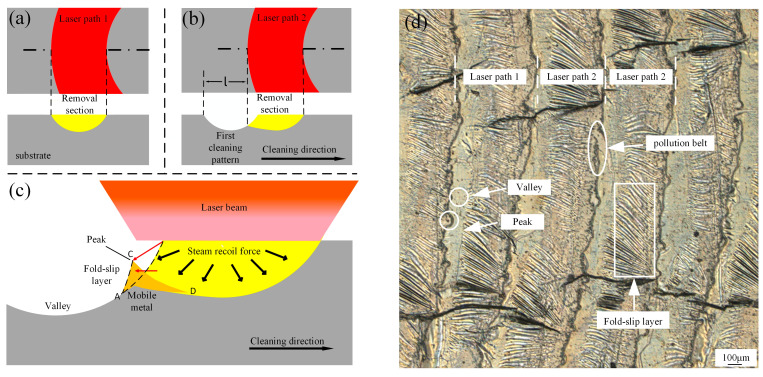
Principle of surface morphology formation of oscillating laser: (**a**) first laser path; (**b**) second laser path; (**c**) principle of surface morphology; (**d**) Macroscopic morphology explanation.

When the spot overlap ratio r > 50%, the previous scanning path is overlapped by the subsequent path due to the Gaussian distribution of the laser energy, as illustrated in Figure 7. During laser cleaning, the first path, shown in Figure 7a, results in the evaporation of the lower yellow portion of the material. When the laser is redirected to continue scanning, as depicted in Figure 7b, the distance between the two paths l represents the cleaning path spacing. If l is too large, the material surface may develop a hill-like undulation, with the highest points referred to as crests and the lowest points as troughs. The formation mechanism of these features is explained in Figure 7c. Due to the Gaussian distribution of laser energy, when the path overlap rate is low, the former scanning path does not fully cover the latter. As the material is heated to a molten state, the flowing liquid metal, influenced by the laser vapor recoil and protective gas flow, forms a pleated slip layer in the AC region. This layer acts similarly to isolation bands, collecting contaminant fragments after evaporation and fragmentation, resulting in the black strips observed in Figure 7d. During subsequent laser sweeps, the same principle applies, with the molten metal forming a pleated slip layer in the CD region, and so on. This process causes pollutants to accumulate in these areas, which is the primary reason for the formation of the black bands.

The cleaning speed v = 1.8 m/min is relatively low, resulting in a high beam path overlap rate r. Consequently, the preceding and subsequent beam paths overlap, and the time interval between two laser encounters is only 3 ms. Due to the low surface tension and good fluidity of the aluminum alloy, the molten metal is rapidly pushed to the sides by laser vapor recoil and solidification. As a result, the contaminants at the crest are repeatedly cleaned multiple times, leading to the formation of an irregular wave-like pattern. However, as the same area is cleaned multiple times, the input of laser energy increases the susceptibility of the area to oxidation and compromises the surface flatness. When the cleaning speed is increased to 3.6 m/min and 4.5 m/min, the energy density per unit area decreases, preventing complete melting of the base material. This results in the formation of banded contaminants at the wave crests without the development of a wave-like morphology.

It is evident that, at two distinct cleaning speeds, the cleaning effectiveness on the material surface initially improves and subsequently diminishes with increasing cleaning power. At cleaning speeds of v = 1.8 m/min and v = 4.5 m/min, the surface quality post-cleaning indicates that preliminary cleaning powers of 3000 W and 3300 W, respectively, yield superior surface morphology. At these two cleaning speeds, the laser power required to achieve optimal cleaning results exhibits minimal variation, as oscillating laser cleaning is primarily influenced by the spot line speed, which is determined by the oscillation frequency and amplitude. At three different cleaning speeds—v = 1.8 m/min, v = 3.6 m/min, and v = 4.5 m/min—the path overlap rates are 69.7%, 39.4%, and 24.2%, respectively. These varying overlap rates result in different distances l between the two paths.

**Figure 8:**
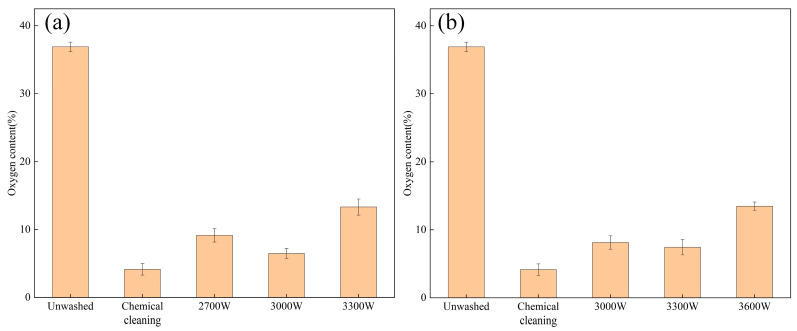
Surface oxygen content under different cleaning speeds: (**a**) v = 1.8 m/min; (**b**) v = 4.5 m/min.

EDS analysis of the surface oxygen elements of 6061 aluminum alloy at two different cleaning speeds was conducted, and the results are presented in Figure 8. Under similar laser cleaning power conditions, the overall cleaning effect at a speed of v = 1.8 m/min was inferior to that at v = 4.5 m/min. At these two different cleaning speeds, the oxygen content of the elements on the surface with the best cleaning effect was 8.48% and 6.44%, respectively. The results indicate that, at a lower cleaning speed, residual pollutants remain at the wave crest, although their impact on the overall oxygen content is not significant. Conversely, at a higher cleaning speed, the overlap rate decreases, resulting in fewer cleaning passes per unit area, which leads to a poorer cleaning effect.

### 3.3. Effect of Oscillating Laser Cleaning on the Quality of Post-Weld Formation

To investigate the impact of laser cleaning on weld formation quality and cross-sectional porosity, three surface treatment methods were employed: untreated, chemical cleaning, and laser cleaning. The parameters for laser cleaning are presented in Table 6.

**Figure 9:**
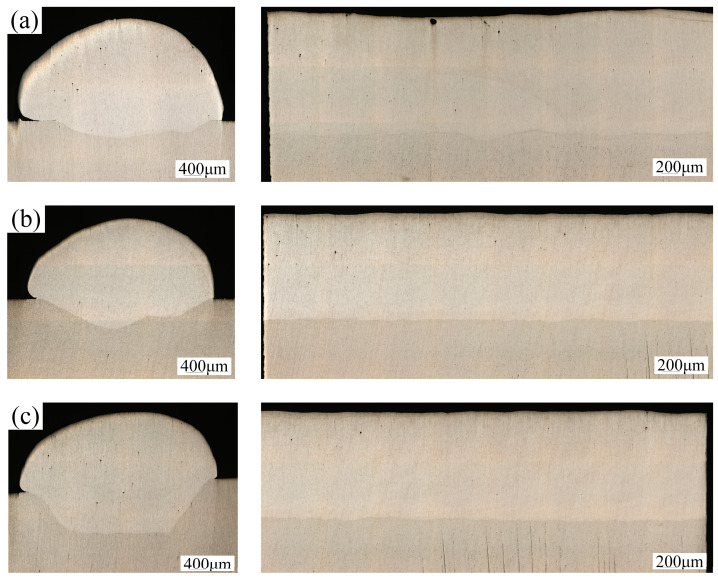
Weld cross-section morphology: (**a**) unwashed; (**b**) chemical cleaning; (**c**) laser cleaning.

The cross-section following welding under the three treatment methods is illustrated in Figure 9. Among the three distinct surface treatment processes, the maximum depth of fusion is achieved after laser cleaning. This outcome is attributed to the pre-heating effect on the material surface induced by laser cleaning, as well as the removal of contaminants such as oxide films, which reduces laser light reflection and enhances energy utilization. As observed in the post-cleaning morphology of the material surface in the preceding section, laser cleaning produces a ring-shaped pattern on the surface. The presence of this pattern increases the surface roughness, thereby enhancing laser absorption to some extent. Subsequently, the porosity under the three surface treatment processes was calculated to be 3.6%, 1.1%, and 1.2%, respectively. This represents a 66.7% reduction in porosity after laser cleaning compared to the cross-section in the untreated state. In addition to achieving a greater melt depth, the porosity following laser cleaning is comparable to that achieved after chemical cleaning, indicating a superior cleaning effect. Following chemical and laser cleaning, welding experiments should be conducted immediately to prevent secondary contamination.

**Figure 10:**
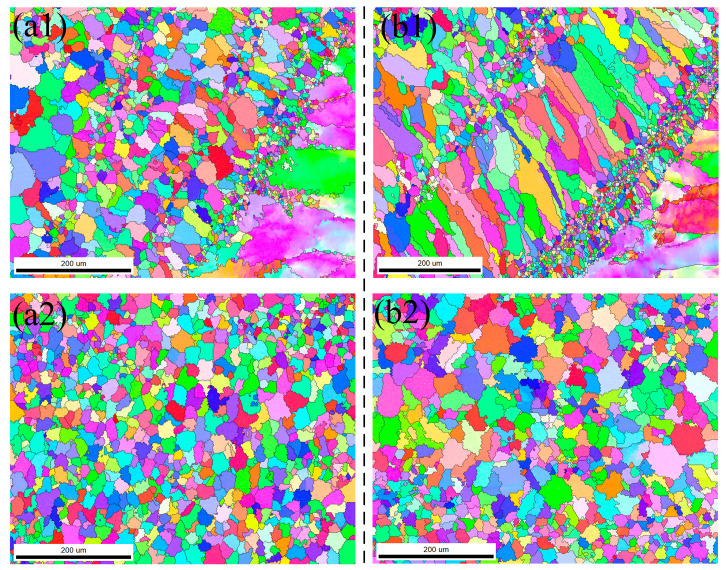
Grain distribution under two cleaning methods: (**a1**) Heat-affected zone under chemical cleaning; (**a2**) Weld center area under chemical cleaning; (**b1**) Heat-affected zone under laser cleaning; (**b2**) Weld center area under laser cleaning.

EBSD was employed to examine the grain size and distribution in the heat-affected zone and the weld center following chemical cleaning and laser cleaning, respectively. As illustrated in Figure 10(a1), the size of the columnar crystals near the heat-affected zone after chemical cleaning is notably different from that observed after laser cleaning, with the former being smaller. However, the distribution of the columnar crystals is less homogeneous compared to laser cleaning, and the growth direction is relatively disordered. In contrast, Figure 10(b1) demonstrates a more banded or continuous grain distribution. The formation of this structure can be attributed to the thermal effects of laser cleaning, which elevate the surface temperature of the material and reduce the temperature gradient, thereby influencing the direction and duration of grain growth. Consequently, laser cleaning exhibits a higher temperature gradient than chemical cleaning and allows sufficient time for grain growth and alignment at a slower cooling rate, resulting in the observed morphology. As depicted in Figure 10(a2,b2), following laser cleaning, the grain size in the center region increases compared to the uncleaned sample. The grain size is primarily determined by the cooling rate of the material; a faster cooling rate results in smaller grains. After cleaning, the cooling rate decreases, prolonging the presence of high temperatures, which facilitates grain growth.

### 3.4. Cleaning Effect of Oscillating Laser on Weld Surface

**Figure 11:**
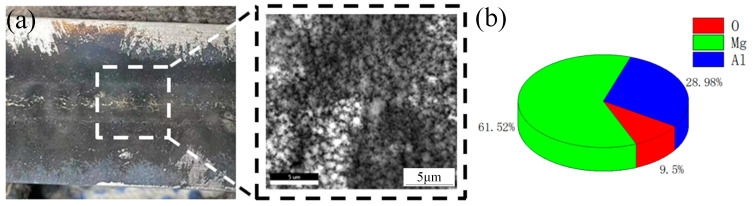
Aluminum alloy welding black ash and composition: (**a**) black ash morphology, (**b**) elemental composition of black ash.

In the aluminum alloy welding process, surface contaminants frequently form on the weld. As illustrated in Figure 11, these contaminants appear as black particles distributed around the weld. EDS analysis reveals that the primary component of these particles is Mg, constituting 61.52% of the composition. Microscopic examination shows that the contaminants exhibit a flocculent, fluffy structural morphology.

**Figure 12:**
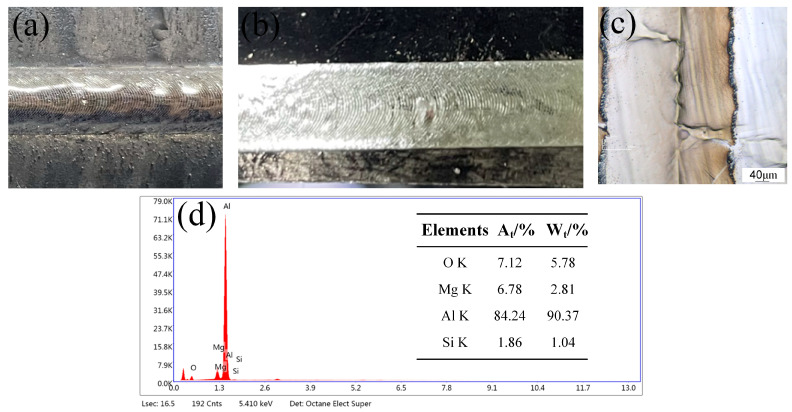
Cleaning effect of pollutants after welding: (**a**) surface after welding, (**b**) laser cleaning of surfaces after welding, (**c**) surface morphology after cleaning, (**d**) EDS analysis results.

The parameters of the laser cleaning process are presented in Table 6, and the removal of post-weld contaminants at and around the weld is illustrated in Figure 12.

Following oscillating laser cleaning, the treated surface exhibits no visible residual contaminants. As illustrated in Figure 12d, the surface morphology demonstrates effective cleaning. EDS analysis reveals significant reductions in the concentrations of Mg and O elements on the material surface, measuring 6.78% and 7.12%, respectively. These values approach those of the cleaner material surface, indicating successful cleaning outcomes.

In summary, continuous laser cleaning is primarily employed for large-scale and thick contaminant removal, yet its effectiveness in cleaning fine structures remains suboptimal. In the future, composite laser cleaning, which integrates the advantages of both continuous and pulsed lasers, is expected to effectively address these limitations. This approach not only enhances cleaning efficiency but also minimizes thermal damage to the substrate.

## 4. Conclusions

In this study, surface contaminants (such as oil stains, metal oxides, and post-weld black ash) on 6061 aluminum alloy were removed using a continuous high-power fiber laser. The research aimed to investigate the feasibility of high-efficiency laser cleaning of 6061 aluminum alloy by varying the laser power and cleaning speed of the continuous high-power fiber laser. Additionally, the morphology, elemental composition, grain size, and distribution of the cleaned samples were analyzed. The following major conclusions were drawn:

1. When the cleaning speed is constant, the oxygen content exhibits a trend of initially decreasing and then increasing with the rise in laser power. Excessive power results in severe ablation and secondary oxidation of the material surface, causing the cleaning effect to diminish. The optimal cleaning effect was achieved at a laser power of P = 3300 W and a cleaning speed of v = 3.6 m/min, with a surface oxygen content of 5.39%.
2. At lower cleaning speeds, the high beam overlap rate results in excessive overall heat input, causing wavy splattering at the edges of the laser scanning path. This phenomenon leads to secondary oxidation, even though the same cleaned area is scanned multiple times.
3. Under the optimal process parameters (laser power P = 3300 W, cleaning speed v = 3.6 m/min), high-power oscillating laser scanning cleaning effectively removes post-weld black ash and reduces the concentrations of Mg and O elements on the weld surface from 61.52% and 9.5% to 6.78% and 7.12%, respectively. Porosity significantly decreased following laser cleaning, which also enhanced the depth of fusion. However, it resulted in an increase in grain size.

## Figures and Tables

**Table 1:** YLS-4000 optical characteristics.

Parameters	Values
Wavelength, nm	1070 ± 5
Feed fiber core diameter, µm	50
Beam parameter product, mm × mrad	2
Minimal process fiber core diameter, µm	100

**Table 2:** The 6061 welding wire chemical composition (wt %).

	Mn	Mg	Zn	Cr	Ti	Si	Fe	Al
6061	0.15	1.0	0.25	0.2	0.15	0.6	0.7	96.95

**Table 3:** The 6061 mechanical properties.

	Tensile Strength (Mpa)	Yield Point (Mpa)	Elongation (%)
6061	260–310	240–270	≥12

**Table 4:** Laser cleaning process parameters.

Parameters	Values
Laser power, W	1200–3900
Cleaning speed, m/min	1.8, 3.6, 4.5
Defocusing amount, mm	0
Oscillation range, mm	5
Oscillation frequency, Hz	300
Oscillation track	Circle

**Table 5:** Laser welding process parameters.

Parameters	Values
Laser power, W	2300
Welding speed, m/min	2
Feeding speed, m/min	4
Defocusing amount, mm	0
Oscillation range, mm	2
Oscillation frequency, Hz	150
Oscillation track	Circle

**Table 6:** Laser cleaning parameters.

Parameters	Values
Laser power, W	3300
Cleaning speed, m/min	3.6
Defocusing amount, mm	0
Oscillation range, mm	5
Oscillation frequency, Hz	300
Oscillation track	Circle

## Data Availability

The original contributions presented in this study are included in the article. Further inquiries can be directed to the corresponding author.
